# The Influence of Yarn and Weave Structures on Acoustic Materials and the Effect of Different Acoustic Signal Incidence Angles on Woven Fabric Absorption Possibilities

**DOI:** 10.3390/ma14112814

**Published:** 2021-05-25

**Authors:** Bethalihem Teferi Samuel, Marcin Barburski, Jaroslaw R Blaszczak, Ewa Witczak, Katarzyna Abramczyk

**Affiliations:** 1 Faculty of Material Technologies and Textile Design, Institute of Architecture of Textiles, Lodz University of Technology, 116 Zeromskiego, St., 90-924 Lodz, Poland; bethalihem.samuel@dokt.p.lodz.pl (B.T.S.); 198304@edu.p.lodz.pl (K.A.); 2 Faculty of Mechanical Engineering, Institute of Turbomachinery, Lodz University of Technology, 219/223 Wolczanska St., 90-924 Lodz, Poland; jaroslaw.blaszczak@p.lodz.pl; 3 Lukasiewicz Research Network-Textile Research Institute, 5/15 Brzezińska St, 92-103 Lodz, Poland; ewitczak@iw.lodz.pl

**Keywords:** woven fabric, polyester fiber, sound environment, acoustic barrier

## Abstract

Utilizing textile-based acoustic materials can be considered basically from two points of view. First, it may be used as a sound absorbing material. Second, it may be used as a decoration that gives the surrounding area a new artistic appearance. To improve the acoustic possibilities of any woven fabric, it is necessary to study the influences of yarn characteristics and the internal structures of weave interlacement. To understand the impact of the yarn on the fabric, the samples were prepared using only polyester fiber as textured, twisted, and staple yarns. Regarding this experiment, the basic weave’s structure type, such as plain, rib, sateen, and twill, were used. Overall, 16 woven fabrics were prepared. The investigation was performed in the range of low to medium acoustic frequencies. The experiments were conducted in an anechoic chamber. Compared to other yarn types, fabrics formed from textured polyester yarn had higher sound absorption properties. Moreover, the observed results show that the different incidence angles of acoustic signals influence the measured sound absorption properties of a textile.

## 1. Introduction

Noise absorption materials are categorized based on resonance absorption, membrane absorption, and porosity absorption. Textile-based materials are classified as porous sound-absorbing materials [1,2].

Porous materials are formed from fibrous materials, composites, and sponges with a small internal diameter of pores, interconnected channels, and cracks or cavities distributed uniformly or randomly throughout the material. The characteristic of the porous structure allows the entrance of sound waves into the material. The friction of sound waves against the pore walls or solid threads enable conversion of the acoustic energy into heat energy which causes the reduction of sound energy. According to the results of other authors, porous materials have good noise absorption properties, particularly in the high-frequency wavebands [3,4].

Woven fabrics are fibrous materials and classified as porous material. Observed by other researchers [5,6], the effectiveness of woven materials for the application of sound absorption is not as effective as nonwoven materials. Specifically, some studies [7,8] show there are problems with low frequency sound absorption for the cases of many porous materials including textiles. However, sometimes unexpected results were observed in specific narrow ranges of these wavebands concerning such materials [9,10].

The attenuation of the noise by woven material depends on the parameters such as the thickness of the fabric, yarn type and characteristics, interlacement type (weave structure), weaving process parameters, and amount of porous space. The nature of the fiber type influences the sound absorption of any material and the sound absorption is enhanced by a lower yarn linear density and finer yarns, as described in [6,11]. Segura-Alcaraz et al. [5] explain that a jute material has better sound absorption properties than other synthetic materials. Moreover, sound reduction using jute fibers at lower frequencies is more effective in comparison to higher frequencies, besides other yarn characteristics, as mentioned in [12]. PES (polyester) textured yarn has the properties of enhanced volume and an improved covering performance for sound absorption material. Regarding the research paper of Soltani and Zerrebini [1], a plain weave structure reduces noise better than other weave types due to the fabric’s higher density, shorter free-float, higher interlacement, and higher crimping yarns. To compare to a plain weave, twill fabric has a higher number of thread floats and a lower number of interlacing points. The plain-weave fabrics show stability due to the higher interlacement points of weft and warp yarns.

Additionally, the air permeability performance is much lower than other weave types [10,13,14,15]. Better sound absorption properties were observed in a sateen weave structure as opposed to double cloth and back weft weaves [6]. This paper also stated that the honeycomb fabric structures have lower sound absorption properties due to their internal open and low-density structures. According to article [16] adding the back air gap increased the honeycomb fabric’s sound absorption capacity, particularly at low frequencies.

Additionally, the structure and characteristics of porous fabrics affect the amount of sound that can be absorbed. Woven fabric is formed with two types of porosity: macro and micro-porosity. Micro-porosity is created by a gap between fibers in the yarns, and macro-porosity is formed between the yarn strands. It is primarily determined by the degree of interlacement and the weave type. Regarding porosity, Havlova states [12] that the possibility of predicting the approximate permeability value depends on the linear density of the yarns used and the diameter of the pores (one inter-yarn). The size of the pores of woven fabric also may be deformed and change their shape due to airflow, but this depends on the degree of the interlacing of the yarns. Furthermore, airflow can cause floating yarns, which can result in the formation of new pores on the fabric surface [17,18].

This article aims to examine the effect of yarn and weave characteristics on the sound absorption properties of materials. A total of sixteen woven samples were prepared and used exclusively for this study. Utilizing various yarn characteristics, filament counts, and weave structures for sample preparation enable us to better understand the role of yarn and fabric properties in textile-based acoustic materials. Correlations between weft and warp density, the effect of yarn twist and hairiness, fabric thickness, mass per unit area, and the air permeability of fabrics were analyzed in relation to the results of acoustic tests. Acoustic tests were conducted in an aeroacoustic anechoic chamber [19,20]. Taking advantage of the ability to measure the sound absorption capacity of fabrics from various angles, the fabrics were measured at a 45-degree angle. The results of the responses of the woven fabrics to sound transmitted in different angle directions demonstrate the need for more precise applications of textile-based noise reduction materials in the future.

## 2. Materials

**Figure 1:**
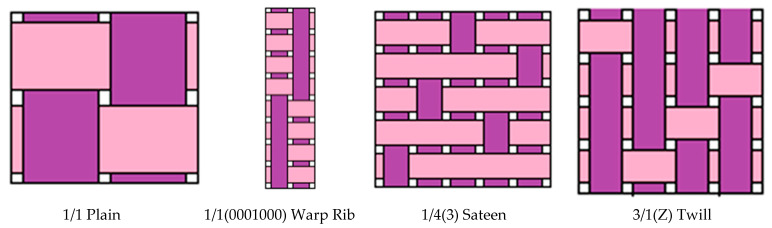
Selected fabric structure interlacement.

**Figure 2:**
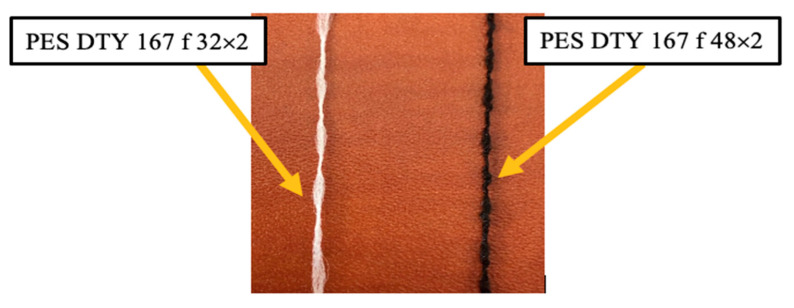
Polyester textured yarn type.

During this experiment, 16 woven fabrics were used. Twelve fabrics were woven in the Lukasiewcz Research Network-Textile Research Institute using a Sample Dobby loom SL 8900S (CCI Tech Inc, Lodz, Poland) with 8 harness frames and 1500 warp yarns for each weave structure. Polyester yarn was used as a raw material in both directions as a warp and weft. Polyester (PES) dtex 167 × 2 (f 32 × 2) drawn textured yarn (DTY), Polyester (PES) 20 × 2 tex staple yarn, and Polyester (PES) dtex 334 f 32 × 2, S95 twisted yarn were used for sample preparation. Four different basic weave patterns (Figure 1), namely, plain, twill, rib, and sateen structures, were prepared from each polyester yarn type (total 12 fabrics). Additionally, four similar basic weave patterns were created using a different weaving machine; a Picanol Gamma loom with a Jacquard machine, in the Institute of Architecture of Textiles in the Lodz University of Technology. Samples were created using PES textured yarn dtex 167 f 32 × 2 for the weft and PES textured yarn dtex 167 f 48 × 2 for the warp (Figure 2). Fabric parameters set and yarn types are presented in Table 1.

## 3. Description of Methodology

Along with the acoustic absorption efficiency tests, the physical properties of the yarn and woven fabrics were examined, as well as their air permeability. The experiments of the physical properties were conducted to deeply understand the actual behavior and the impact of various yarn characteristics and woven structures on sound barrier material applications.

### 3.1. Physical Characteristics of Yarn and Fabrics

The measurements of the yarn characteristics were performed on polyester-drawn textured yarn, polyester staple yarn, and polyester twisted yarn. The measurements were executed according to standards. The yarn twist was conducted according to ISO 2061–2010 [21] under normal climatic conditions. According to ISO 2649-1974 [22] (temperature 20 °C, and relative humidity 69%), yarn hairiness and yarn evenness were examined using USTER TESTER 3. Table 2 describes each yarn characteristic with different levels of yarn twist, yarn hairiness, and yarn evenness values.

Fabric characteristics that help to predict the performance of the fabrics for the application barriers were examined. The warp and weft densities were determined. Fabric thickness tests were performed with the measurement area of 20 cm^2^ and at a pressure of 1 kPa according to PN-EN ISO 5084:1999 [23] (temperature 20 ± 0 °C, and relative humidity 65 ± 5%). Yarn crimp in the fabric was calculated according to ISO 7211-3:1984 [24]. Fabric mass per unit area was tested according to PN-ISO 3801:1993 [25] (temperature 21 ± 0 °C, and relative humidity 66 ± 5%). Measured construction parameters are presented for the 16 woven fabric samples in Table 3.

Plain II (TPII), rib II (TRII), sateen II (TSII), and twill II (TTII) are the terms used to describe fabrics created on a Jacquard loom using textured yarn (II). Fabrics were produced on a Sample Dobby loom SL 8900S (CCI Tech Inc) with textured yarns (I) and are described as plain I (TPI), rib I (TRI), sateen I (TSI), and twill I (TTI). Plain (SP), rib (SR), sateen (SS), and twill (ST) fabrics were fabricated using staple yarn, and plain (TwP), rib (TwR), sateen (TwS), and twill (TwT) structures were prepared using twisted yarn.

### 3.2. Air Permeability Test

All sixteen samples were subjected to an air permeability test. Regarding each sample, a 20 cm^2^ surface area on the unwrinkled surface of the fabric was measured, and air pressure drops of 100 Pa were applied selectively to ten different locations on the sample. All procedures and calculations were completed according to ISO 9237:1995 [26]. Each material’s air permeability was determined using the Fx 3300 air permeability tester following a 24-h acclimatization period in normal climatic conditions (temperature 20 °C, relative humidity 65%). The tests were executed in the Department of Materials Science, Commodity Science and Textile Metrology laboratory at the Lodz University of Technology (Lodz, Poland).

To calculate the air permeability, the following formula was used:

Q = (qv/Ap) × 167

where

Q = The velocity of air flow perpendicular to a sample (mm/s)
qv = The average mean of the air flow (dm^3^/min or liter/min)
Ap = Test area of the specimen (cm^2^)
167 = Conversion factor from (dm^3^ or liters/min and cm^2^ to mm/s)

### 3.3. Acoustic Tests

**Figure 3:**
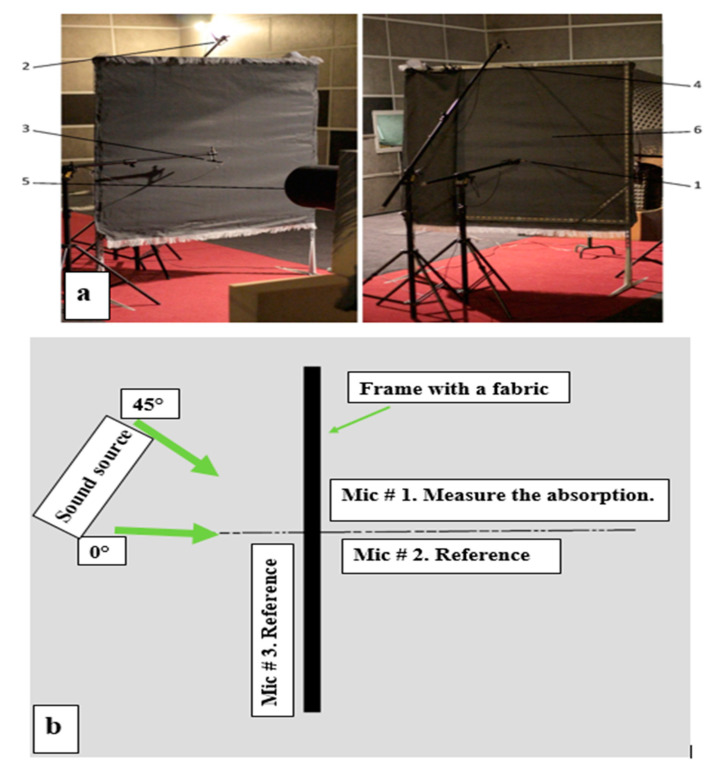
(**a**) Test stand: 1—microphone #1, 2—microphone #2, 3—microphone #3, 4—frame, 5—the sound source, 6—tested fabric; (**b**) Top view of the measurement area.

Sound absorption tests were conducted in a free-field environment inside the aeroacoustic anechoic chamber in the Laboratory of Aeroacoustics of the Institute of Turbomachinery at the Lodz University of Technology (Figure 3a). Prior to beginning the main tests, the sound levels were measured without any textile for the purpose of validating the measurement system and for reference purposes. Subsequently, the fabrics were tested in the selected frequency ranges according to international standards ISO 26101:2012 [27]. The temperature was 22 °C, and the relative humidity was 65%, and the frequency ranges were 63, 125, 250, 500, 1000, and 2000 Hz.

Sound level drops were measured for all samples according to the chosen frequency ranges and at different angle directions. The first test occurred at the center of the fabric by setting the sound source directly in front of the first microphone at the incidence angle of 0° (Figure 3b). During the second test, the sound source was placed at 45°. Regarding each case, a distance of 1 m was maintained between the sound source and the fabric surface. The distance between the fabric and microphone #1 was 0.1 m. Microphone #2 was set vertically at the same line above microphone #1 at 0.1 m above the frame with the fabric, and was used for checking the reference conditions. Microphone #3 was placed on a horizontal line to microphone #1 and the center of the sound source output plane and 0.1 m apart from the fabric. The fabric on the frame was in the middle of microphones #1 and #2. According to the standards, the reference sound pressure level p_0_ was 20 μPa. Details of the anechoic chamber experimental procedures and explanations were described in previous papers [6,8,9,19].

## 4. Air Permeability Test Results

### 4.1. The Influence of Yarn Type on Air Permeability Results

**Figure 4:**
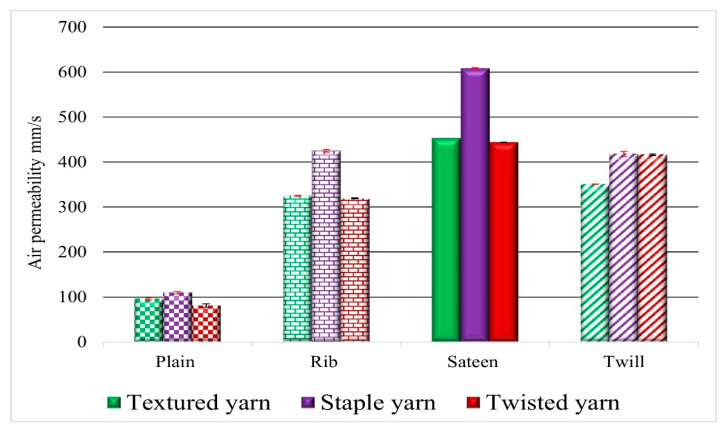
Overall air permeability test results.

Three types of yarns were applied to observe the efficiency of air permeability in woven fabric. The first was the drawn textured yarn (I) shown in Figure 4 (green color). It is characterized as a multifilament yarn, with high bulk due to less twist, soft, and crimp properties. The second was a staple polyester yarn (purple color), described as thin, hairy, and having a higher twist than the other yarns. The third was twisted yarn (dark red color). The number of twists imparted to the thread was between the textured and staple yarns. Thus, the thin and thick places formed throughout the strand give a higher yarn evenness value (Table 2). Low air permeability results were obtained from the overall results with twisted yarn (dark red bar), except for the twill fabric. The second comparable results were obtained with fabrics formed from textured yarn (green bar) in all types of weave structures, and show the second lowest air permeability result. The third result is for staple yarn, which exhibits a high air permeability. (Figure 4).

### 4.2. The Influence of Fabric Structure on Air Permeability Results

Shown in Figure 4, the patterns in the graph depend on the fabric characteristics, such as a plain fabric structure (rectangular 1/1 pattern) and a twill fabric’s distinguishing characteristics (diagonal pattern). The sateen weave on one side is shiny and smooth while, on the other side, it forms a dull effect, and the rib structures are the derivatives of plain fabrics with a floating of yarn in the structure. The structure design was chosen by considering the amount of porous space created by the interlacement and the floating of threads without interlacement. The influence of the inter-geometrical structure of the fabric determines the air permeability directly or indirectly, and the acoustic properties. As the number of interlacements increases, the stiffness of the fabric increases simultaneously and prevents the yarn from floating. Yarn floating is a term that refers to a warp or weft yarn that floats over two or more opposing yarns in the fabric structure without interlacing.

The plain fabric structure formed from all PES has a low air permeability result compared to the other fabric structures. Plain fabrics initially have a high interlacement pattern. Accordingly, the interlacement points are higher than the other structures and cause the stiffness behavior of the fabric. This leads to the resistance to air pressure applied by the air. The results for the rib and twill fabric structures are similar. Specifically, rib 1/1 fabrics formed from textured yarn (TRI) and twisted yarn (TwR) have a lower permeability compared to rib (SR) which is formed from staple yarn.

## 5. Sound Absorption Test Result

### 5.1. Influence of Yarn Type and Fabric Performance on Sound Absorption

**Figure 5:**
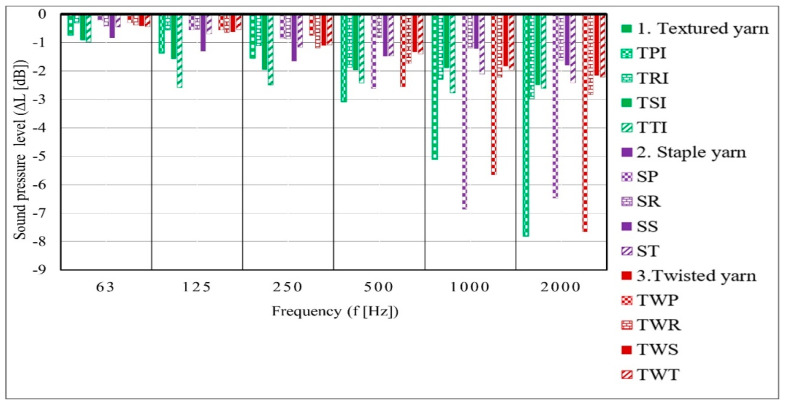
Sound pressure level drop versus frequency range.

The sound absorption measurements for all fabrics were performed for 63, 125, 250, 500, 1000, and 2000 Hz frequency ranges (Figure 5).

The selected woven fabric structure has a low sound absorption performance at low-frequency ranges (Figure 5). However, the sound absorption performance increases with high-frequency fields. The effectiveness of each fabric type on the sound absorption performance depends on the yarn types. The staple yarn was thin due to a high number of twists per meter compared to other yarn types. While the number of twists imparted increases, the cohesion between fibers also increases. Therefore, the porous space between the fibers becomes smaller, and the yarn becomes thin. The possibility of porous space between the interlacement on the fabric surface increases proportionally as the yarn thickness increases, but this phenomenon may vary depending on the weave structure type. The results indicate that the hairiness of the staple yarns on the fabric has no effect on the sound level drop. Additionally, as an obtained result, the performance of the sound pressure level drop in such fabrics is low. The small number of turns per meter imparted to the twisted PES yarn gives it a higher surface evenness throughout the yarn. The result shows that the sound absorption by fabrics formed from the twisted yarn is higher than the fabrics formed from the staple yarn. The bulkiness of the textured yarn (I) permits the entrance of sound waves between the fiber strands. This phenomenon may increase the chance of the absorption of sound energy between the fibers. To compare, the overall sound level drop indicates that fabrics made of textured yarn (I) have a significantly higher sound absorption than other fabrics.

The performance of each weave structure in the measured frequency ranges is different. First, at low-frequency ranges of 63–250 (Hz) the TTI weave structure has higher absorption properties compared to other structures. Second, the TSI fabric in the same range shows an intermediate result. Concerning a similar range, the SS structure has the highest absorption and is followed by the ST fabric. The fabrics formed from twisted yarn have the smallest sound level drop at a low frequency between 63 to 250 f(Hz) in comparison to fabrics formed from staple yarn and textured yarn.

Accompanying an increasing frequency (500 Hz up to 1000 Hz), the TPI, SP, and TwP fabric structures have higher sound pressure level drops. Specifically, SP fabric has a higher sound absorption property at the frequency of 1000 Hz. Other than plain fabric, the twill structures follow with the second highest absorption at 1000 f(Hz) except for the TwT sample. Seen at 2000 f(Hz), the plain weave structure shows higher sound reduction properties than other structures in all yarn types.

**Figure 6:**
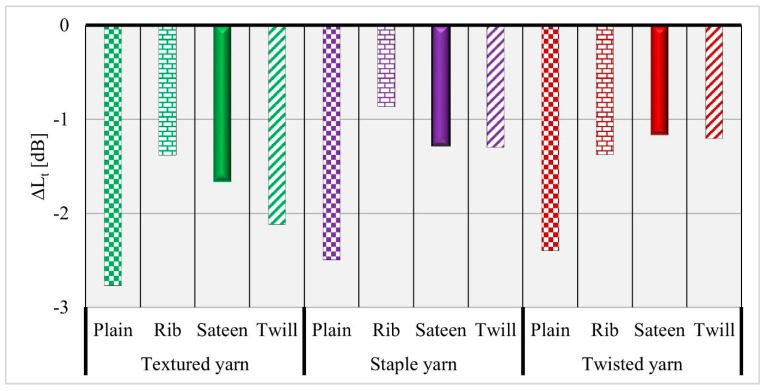
Overall comparison of total sound pressure level drop versus yarn type and fabric structures.

The total sound pressure level drop results show (Figure 6) that the textured yarn (I) (green colors) has the highest absorption potential, and it may be concluded that the bulkiness of the yarn increases its ability to absorb sound energy between the fibers. Generally, the fabrics formed from textured yarn (I) in all measured frequencies show a higher sound absorption potential. Conversely, materials created from staple and twisted yarns have comparable outcomes at a low frequency. However, for fabrics with twisted yarn, the absorption potential increases when the frequencies increase. The measurements were performed for every selected frequency range and based on the results the overall acoustic pressure was calculated [20].

### 5.2. The Influence of Yarn and Weave Parameters on the Reduction of Air Permeability and Sound Absorption Potential

To investigate the effect of yarn characteristics on the sound absorption and air reduction of fabrics, samples were made exclusively from PES textured yarn with varying yarn set densities and filament counts.

**Figure 7:**
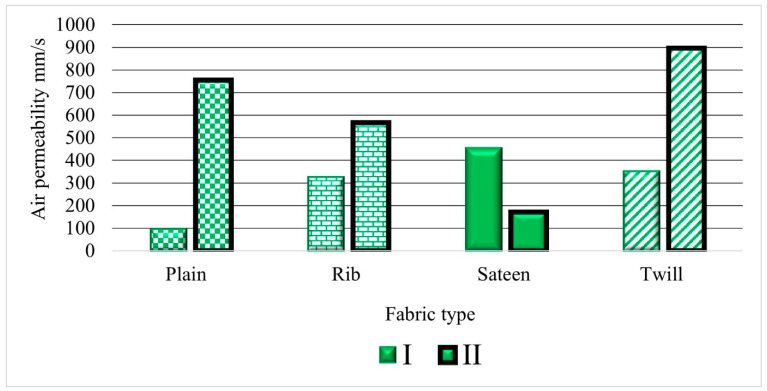
Comparisons based on yarn and weave parameters. Textured yarn (I) and textured yarn (II).

The findings indicate that the fabrics produced using textured yarn (I) are completely distinct from those produced using textured yarn (II). The result demonstrates (Figure 7) that TPI has a low air permeability performance compared to TPII. The weft density of the TPI is higher than the TPII. The other fabric structure, TSII, has a low air permeability. The situation could occur because TSII (33/30) has a higher density of ends per pick than TSI (34/20). The increase of the per centimeter yarn count proportionally increases the fabric’s density. This raises the possibility of decreasing the fabric’s air permeability.

Conversely, TTI and TTII fabrics have a similar density of ends per pick. However, the TTI has higher air reduction properties than the TTII structure. Except for the density, the thickness and crimp percentage of the TTI fabric is higher than the TTII fabric structures. The other reason is that the fabric weave design influences the result of the absorption, such as the sateen weave structure which has a long free float yarn on the surface of the fabric. The fibers in the yarn may move easily from their position while air pressure is applied, and this gives a possibility to let the air flow through the material.

**Figure 8:**
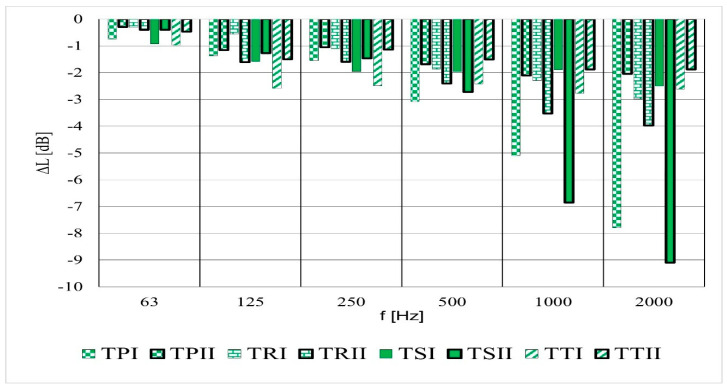
Sound pressure level drop versus frequency; textured yarn (I) and textured yarn (II).

The comparison of the sound absorption of fabric based on yarn parameters shows that the results for the textured yarn (I) fabrics such as TPI, TSI, and TTI fabric structures at low-frequency ranges (63–125 Hz) show a higher potential than for the textured yarn (II) fabrics (Figure 8).

Conversely, at higher frequency range (1000 Hz and 2000 Hz), the TRII and TSII fabrics structures have significantly higher sound absorption properties.

**Figure 9:**
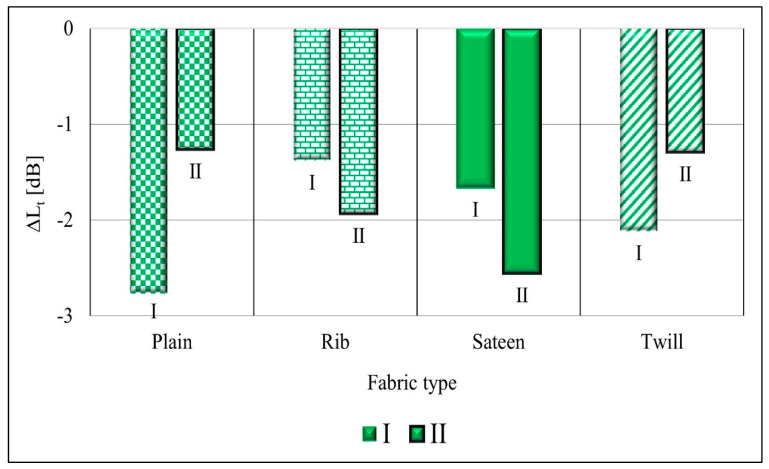
Comparison of total sound pressure level drop between fabrics formed from; textured yarn (I) and textured yarn (II).

Fabrics formed from textured yarn (I) show that the plain structure has the highest sound absorption potential, aside from the twill fabric (Figure 9). The TSII has the highest sound absorption properties at higher frequency ranges. Additionally, the TRII structure is the second highest sound absorption material.

Fabrics formed using textured yarn (I) consist of a warp and weft per filament of 5.2 dtex, and the fabric formed using textured yarn (II) per filament is 3.48 dtex for the warp and 5.2 dtex for the weft. Consequently, the fabrics which were formed from textured yarn (I) had thicker filaments than textured yarn (II). These phenomena increase the possibility of absorption and it can be concluded that the thickness of the filament in fabrics made from textured yarn (I) increases the possibility of friction of sound energy between the filaments. This may account for the high absorption capacities of fabrics made from textured yarn I.

**Figure 10:**
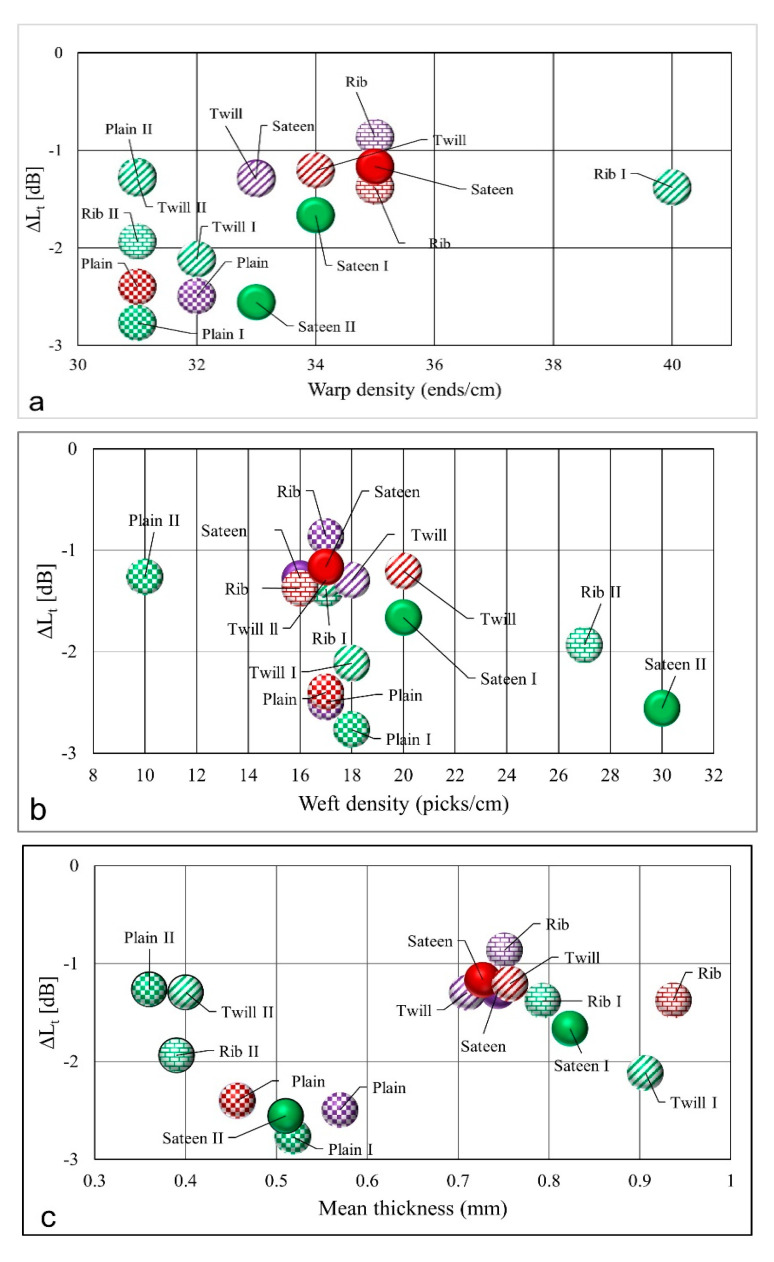

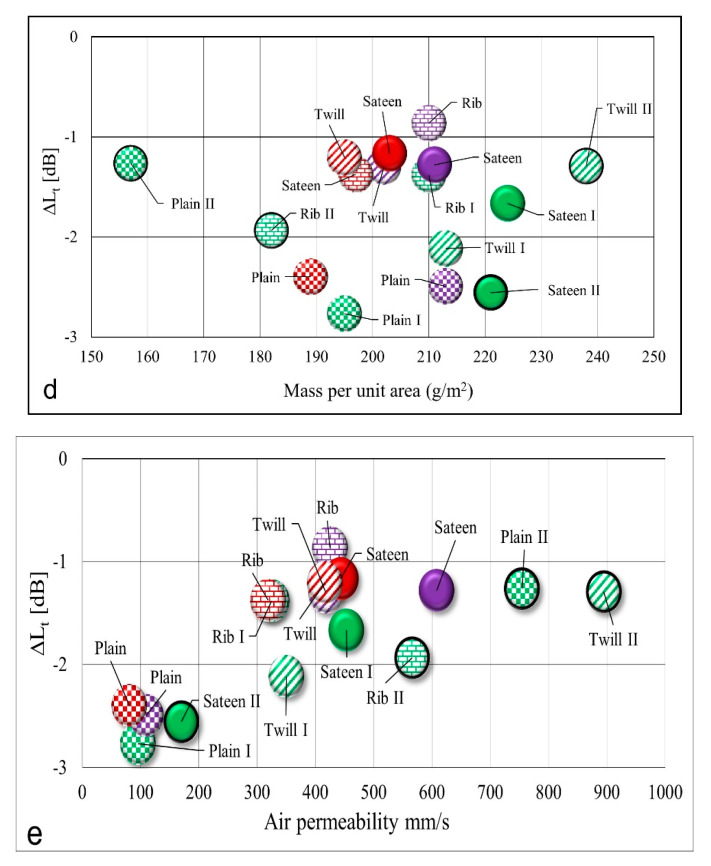
Overall yarn type and fabric properties comparison concerning sound level drop at 0°. (**a**) Illustrates the relationship between the warp density of fabrics and the total sound pressure level drop. (**b**) Presents the weft density of fabrics in relation to total sound pressure level drop. (**c**) Depicts the effect of fabric thickness on fabric total sound pressure level drop. (**d**) Illustrates the effect of fabric mass per unit area on total sound pressure level drop. (**e**) Depicts the relationship between the fabrics’ total sound pressure level drop and air permeability performances.

According to the test results in Figure 10, the plain fabric structure has the highest absorption properties compared to the other structures. The plain fabrics, which are indicated by the green (I), dark red, and purple color with rectangular patterns, have a warp per centimeter between 30 to 34 (Figure 10a) and a weft per centimeter between 16 to 18 (Figure 10b). This indicates a very similar number of ends and picks per centimeter. Shown in Figure 10 (a and b cases), the results vary depending on the total sound absorption level, from highest to lowest, for the following yarn types: textured yarn (I) (plain I), staple yarn, and twisted yarn. TSII (indicated by a shiny green color) is another structure with a high sound absorption result. It has a similar warp per centimeter as plain fabrics, but it has a higher weft per centimeter than all plain fabric structures.

Additionally, TSII has a high density compared to the other structures. The thickness properties do not show any significant variations in this research result. The rib (TwR) structure (Figure 10c) has the highest thickness; however, the sound absorption performance is low. The mass per unit area (Figure 10d) of TTII (green diagonal pattern) is higher than the rest of the fabric structures. Nevertheless, the absorption performance of TTII is low.

The relationship between the air permeability and sound reduction properties is inversely proportional. The lower air permeability result foreshadows the higher sound absorption material. Thus, seen in Figure 10e, as the total sound pressure level drops increase, then the air permeability decreases. The comparison between the yarns indicates that the fabrics which are formed from twisted yarn and textured yarn have comparable low air permeability results. However, the sound drop is higher with the fabric formed from textured yarn. Generally, the comparison depends on weave structure; a low air permeability and high sound pressure level drop were confirmed on plain weave structures and for the second TSII weave structure.

### 5.3. Comparison of the Sound Pressure Level Drop According to Different Angles

**Figure 11:**
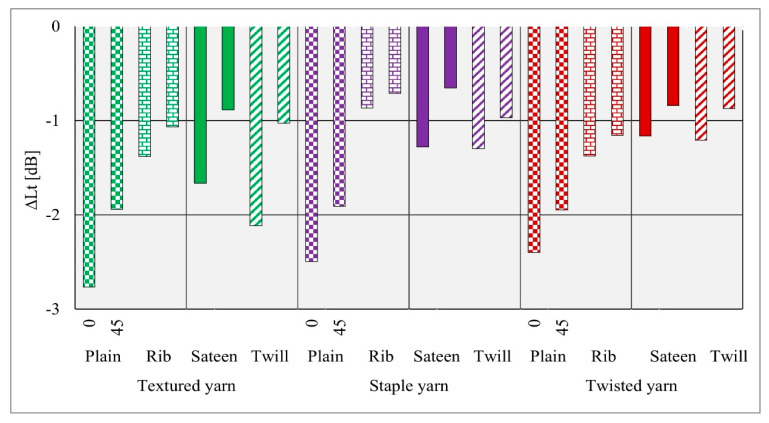
Comparison of sound pressure level drop at the directions of 0° and 45°.

During the tests, it was noticed that the sound absorption property also is influenced by the incidence angle of the sound wave (Figure 11). When the tests took place, the sound source position was changed with respect to the center of the fabric. Hence, the four different fabric structures formed from three yarn types (12 fabrics) revealed different responses according to the sound wave direction. The results for 0° showed higher sound absorption properties than for 45°. When the angle was changed to 45°, all fabrics showed a slightly lower sound absorption potential.

## 6. Summary and Conclusions

This paper presents the influence of yarn characteristics, weave structure, and the effect of different acoustic signal angles on fabric absorption results.

- Regarding comparison purposes, PES staple yarn was thin and had a high number of twists per meter. The amount of twist imparted on the staple yarn may prevent the entering of sound waves into the yarn strand or it lets sound waves to pass directly without any energy exchange between the strands. The fabrics formed from staple yarn also showed such results in the sound attenuation of the fabrics. Conversely, staple yarn hairiness properties do not significantly affect sound absorption and air permeability potential. Generally, materials formed from the staple yarn show a high air permeability and low sound pressure reduction results.
- Low air permeability results also were recorded for all fabrics formed from twisted yarn, except the twill fabric structure. The textured yarn (PES DTY dtex 167 f 32 × 2) has a bulky structure and thick filaments in comparison. The bulkiness enables entrance of the sound waves between the fibers, and the thickness of the filament may increase the chance of sound energy friction with the filaments. This phenomenon may have the potential to dump sound waves between the fibers. High sound absorption results were achieved by all fabrics formed from textured yarn (I).
- The results of measuring in various directions in the anechoic chamber indicate that the acoustic measurements at a 0° incidence angle showed higher sound pressure level reduction properties in all low and medium frequency ranges than those obtained at a 45° incidence angle. These findings provide guidance for the use of such materials in interior applications such as cinema interior wall coverings and conference rooms.
- To obtain the maximum sound absorption potential of the presented materials, the sound source setting position or the incidence angle play decisive roles. Further experiments are in progress, specifically on the relation of the porosity of woven fabrics and the influence of increasing layers on textile acoustic materials.

## Figures and Tables

**Table 1:** Type of yarn and fabric parameters set.

Yarn Types	Weave Types	Sample Name	Set Density Warp/Weft (cm)
Textured	Plain I	TPI (Textured yarn-Plain weave I)	30/16
	Rib I	TRI (Textured yarn-Rib weave I)	30/16
	Sateen I	TSI (Textured yarn-Sateen weave I)	30/16
	Twill I	TTI (Textured yarn-Twill weave I)	30/16
	Plain II	TPII (Textured yarn-Plain weave II)	30/10
	Rib II	TRII (Textured yarn-Rib weave II)	30/25
	Sateen II	TSII (Textured yarn-Sateen weave II)	30/30
	Twill II	TTII (Textured yarn-Twill weave II)	30/16
Staple	Plain	SP (Staple yarn-Plain weave)	32/16
	Rib	SR (Staple yarn-Rib weave)	31/16
	Sateen	SS (Staple yarn-Sateen weave)	31/16
	Twill	ST (Staple yarn-Twill weave)	31/16
Twisted	Plain	TwP (Twisted yarn-Plain weave)	32/16
	Rib	TwR (Twisted yarn-Rib weave)	31/16
	Sateen	TwS (Twisted yarn-Sateen weave)	32/16
	Twill	TwT (Twisted yarn-Twill weave)	31/16

**Table 2:** Physical properties of three different yarn types.

Type of Yarn		Yarn twist/m	Yarn Hairiness	Yarn Evenness		Measured Linear Density of Yarns (Tex)
				Thin/km	Thick/km	
**Textured yarn**		------	------	------		36.62 ± 0.04
**I**	**Warp/Weft** **PES DTY dtex 167 f 32 × 2**					
**II**	**Warp** **PES DTY dtex 167 f 48 × 2** **Weft** **PES DTY dtex 167 f 32 × 2**					
**Staple yarn** **PES 20 × 2 tex**		511.4	7.24	80	34.6	41.16 ± 0.09
**Twisted yarn** **PES dtex 334 f 64, S95**		90.1	------	------	------	36.93 ± 0.01

**Table 3:** Measured construction parameters for 16 woven fabric samples.

Samples Name	Measured Warp Density (Ends/cm)	Measured Weft Density (Picks/cm)	Fabric Thickness (mm)	Crimp %		Mass Per Unit Area, (g/m^2^)
				Warp	Weft	
TPI	31 ± 0	18 ± 0	0.52 ± 0.01	8.7 ± 0.4	0.9 ± 0.1	195 ± 1.5
TRI	38 ± 0	17 ± 0	0.8 ± 0.02	1.9 ± 0.1	17.9 ± 0.7	224 ± 2.8
TSI	34 ± 0.4	20 ± 0.6	0.9 ± 0.03	11.1 ± 0.1	2 ± 0.1	213 ± 2.1
TTI	32 ± 0	18 ± 0.6	0.79 ± 0.02	10.9 ± 0.2	1.8 ± 0.1	210 ± 4.2
TPII	31 ± 0	10 ± 0	0.36 ± 0.01	6 ± 0.1	0.9 ± 0.1	157 ± 1.8
TRII	31 ± 0.7	27 ± 0.5	0.5 ± 0.02	11.4 ± 0.1	0.8 ± 0.1	221 ± 2.4
TSII	33 ± 0	30 ± 0.6	0.4 ± 0.03	6.5 ± 0.2	2.3 ± 0.1	238 ± 2.2
TTII	31 ± 0	17 ± 0.6	0.39 ±0.02	7 ± 0.1	1.2 ± 0.2	182 ± 1.9
SP	32 ± 0.2	17 ± 0.6	0.57 ± 0.01	14.7 ± 0.3	2.3 ± 0.1	213 ± 1.6
SR	35 ± 0	17 ± 0.5	0.74 ± 0.01	1.3 ± 0.1	11 ± 0.2	211 ± 1.5
SS	33 ± 1.1	16 ± 0	0.71 ± 0.03	6.6 ± 0.1	3.2 ± 0.1	202 ± 0.5
ST	33 ± 0.2	18 ± 0	0.75 ± 0.01	7.5 ± 0.3	2.1 ± 0.1	210 ± 3.3
TwP	31 ± 0	17 ± 0.8	0.46 ± 0.01	7.4 ± 0.2	0.3 ± 0.1	189 ± 0.75
TwR	35 ± 0.1	16 ± 0.6	0.73 ± 0.02	3.1 ± 0.2	11.9 ± 0.2	203 ± 2
TwS	35 ± 0	17 ± 1	0.76 ± 0.02	5 ± 0.2	2.5 ± 0.1	195 ± 0.63
TwT	34 ± 0.2	20 ± 1	0.94 ± 0.01	6.7 ± 0.2	1.9 ± 0.1	197 ± 0.8

## Data Availability

The data presented in this study are available by request to the corresponding author.

## Nomenclature

ΔL	sound pressure level drop (dB)
L	sound pressure level (dB)
F	frequency (Hz)
A	amplitude of the acoustic signal (Pa)
P	acoustic pressure (Pa)
